# Stable Positions of Epigenetically Inherited Centromeres in the Emerging Fungal Pathogen *Candida auris* and Its Relatives

**DOI:** 10.1128/mBio.01036-21

**Published:** 2021-07-06

**Authors:** Laura N. Rusche

**Affiliations:** a Department of Biological Sciences, grid.273335.3University at Buffalo, State University of New York, Buffalo, New York, USA

**Keywords:** *Candida auris*, centromere, epigenetic inheritance

## Abstract

Candida auris is an emerging fungal pathogen that is thermotolerant and often resistant to standard antifungal treatments. To trace its evolutionary history, the Sanyal lab conducted a comparative genomic study focusing on the positions of centromeres in C. auris and eight other species from the *Clavispora*/*Candida* clade of yeasts (A. Narayanan et al., mBio 12:e00905-12, 2021). These researchers discovered that these species possess small regional centromeres that are highly stable, having remained in the same syntenic positions for over 100 million years. This stability is remarkable, given the lack of a conserved sequence underlying the centromeres and the relative ease with which other yeasts form neocentromeres. Thus, this work provides an opportunity to investigate the molecular mechanism of centromere inheritance in a genetically tractable and medically important yeast.

## COMMENTARY

Centromeres, the points at which chromosomes attach to the mitotic spindle, promote genome integrity by ensuring equal segregation of sister chromatids upon cell division. However, they are also sites of chromosomal breaks and rearrangements, and this genomic instability is proposed to promote rapid adaptation to stress (1). These seemingly opposing aspects of centromeres have drawn attention to their properties in opportunistic and emerging fungal pathogens, raising the question of whether centromeres drive genomic changes that enhance virulence. A recent study from the Sanyal lab provides insights into this issue by identifying the locations and features of centromeres in the emerging fungal pathogen Candida auris and its relatives (2).

**FIG 1:**
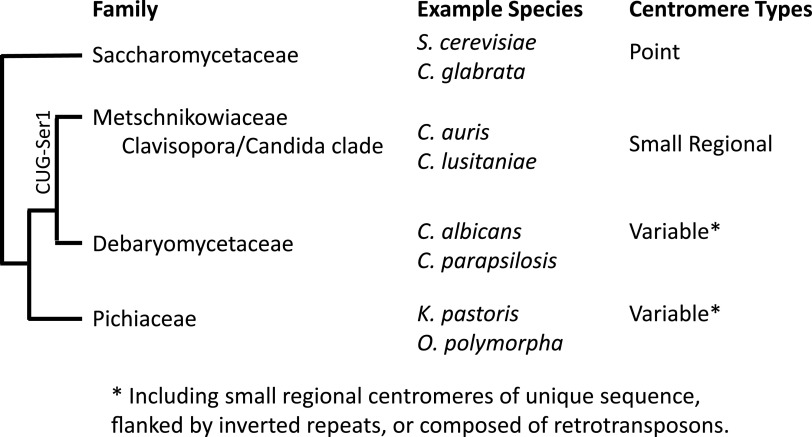
The cladogram indicates the relationships of four yeast families. For each family, representative species and centromere types are indicated.

C. auris was first described in 2009 and is of increasing concern because of its thermotolerance and resistance to commonly used antifungal drugs, leading to high mortality for bloodstream infections (3). C. auris spreads easily in health care settings, where it primarily affects immunocompromised people. There have been hospital outbreaks in four geographic regions: India and Pakistan, Japan and Korea, South Africa, and Colombia and Venezuela. Isolates from a single region are highly similar in genome sequence, suggesting clonal propagation (4). However, there are substantial differences (tens of thousands of single nucleotide polymorphisms) between isolates from the four regions. These observations raise the conundrum of why these genetically distinct clades of C. auris all began causing infections at the same time (5, 6). Compared to other pathogenic *Candida* species, C. auris is most closely related to Candida lusitaniae and belongs to the *Clavispora*/*Candida* clade of the *Metschnikowiaceae* family (Fig. 1).

To identify centromeres in C. auris, the Sanyal group mapped the genomic distribution of the centromere-specific histone, Cse4, using chromatin immunoprecipitation followed by sequencing (ChIP-Seq). Cse4, also known as CENP-A or CenH3, replaces histone H3 in centromeric nucleosomes but is not found in other genomic locations. These researchers identified one site of Cse4 enrichment per chromosome. These presumed centromeres had properties similar to previously characterized centromeres in the related yeast C. lusitaniae (7). Specifically, each sequence was unique, lacked repeated elements, and had a lower GC content than the genome average. In addition, the centromeres did not appear to be flanked by pericentromeric heterochromatin, based on observations that Cse4 occupied most of the intergenic region (2.5 to 3 kb) and that the flanking genes had typical levels of expression.

C. auris has a population structure comprised of distinct clades, each of which appears to propagate clonally. A representative of clade 1 from South Asia was used for the ChIP-Seq experiment. To determine whether centromere positions were consistent in the other clades, the authors examined the loci identified as centromeres in clade 1. Indeed, in all other clades these loci were enriched for Cse4 and depleted of histone H3, indicating that the centromere positions are conserved.

It was possible that the recent emergence of C. auris was facilitated by changes in centromere locations. However, the authors found remarkable conservation of centromere position across the *Clavispora*/*Candida* clade, whose last common ancestor existed over 100 million years ago (8). Five of the seven C. auris centromeres are flanked by the same genes as in C. lusitaniae, and the other two centromeres are flanked on one side by the same genes. In addition, these authors found that these same loci are probable centromeres in three species of the closely related *C. haemulonii* complex (Candida haemulonii, Candida pseudohaemulonii, and Candida duobushaemulonii) and four more distantly related species (Candida fructus, Candida intermedia, Candida blattae, and Candida oregonensis). In all seven species, presumed centromeres were depleted of histone H3 relative to H4, occurred at a trough in GC content, and had unique sequences. Thus, centromeres have been retained at the same syntenic locations for over 100 million years. The only other yeast clade in which such positional conservation has been observed is the *Saccharomycetaceae* family (9), in which centromeres are specified by a particular sequence and hence may be harder to relocate. Two other families (*Debaryomycetaceae* and *Pichiaceae*) display variable centromere organization and position (10) (Figure).

An important topic for future investigation will be how centromere position is stably inherited in the *Clavispora*/*Candida* clade. Unlike the centromeres in the *Saccharomycetaceae* family, there is no indication that centromeres in the *Clavispora*/*Candida* clade are specified by the DNA sequence. In both C. auris and C. lusitaniae, each centromere has a unique sequence (2, 7). Moreover, the Sanyal group found that intergenic regions containing centromeres are evolving more rapidly than other intergenic sequences. Nevertheless, Cse4 has been accurately deposited over the same stretch of DNA each time the genome has been replicated for over 100 million years. This “epigenetic” inheritance is remarkable given the seeming ease with which neocentromeres can form at new locations in species such as C. albicans and C. parapsilosis (10, 11). Epigenetic inheritance of centromeres is widespread across eukaryotes. Although the molecular mechanism is incompletely understood (12, 13), it is known that Cse4 is deposited by a chaperone (Scm3 in yeast and HJURP in mammals). It will be interesting to learn how this chaperone is targeted to C. auris centromeres.

Despite the remarkably conserved positions of centromeres in the *Clavispora*/*Candida* clade, the authors noted several genome rearrangements. For example, a reduction from eight to seven chromosomes occurred in a common ancestor of C. auris and the *C. haemulonii* complex. This occurred through a chromosome fusion, followed by the inactivation of one centromere on what would initially have been a dicentric chromosome (now C. auris chromosome 4). Other chromosome rearrangements, including the above-noted centromere-adjacent synteny breaks between C. auris and C. lusitaniae, were also present in the common ancestor of C. auris and the *C. haemulonii* complex. Thus, a significant reorganization of chromosomes separates this branch from the rest of the *Clavispora*/*Candida* clade.

A more recent set of rearrangements is specific to clade 2 of C. auris. This clade, found in East Asia, is less virulent than the other clades and could theoretically represent an ancestral state. However, the chromosomal configuration of clade 2 differs significantly from that of the *C. haemulonii* complex, whereas the other three clades have more similar chromosomal organizations. Thus, at least with respect to chromosomal organization, clade 2 is derivative rather than ancestral. Several of the observed chromosomal rearrangements in clade 2 occurred within 100 kb of the centromeres. Notably, a 145-kb segmental duplication that includes a centromere has resulted in two apparent centromeres on one chromosome. It will be interesting to determine whether one of these centromeres has been inactivated, as expected to preserve accurate segregation. Several other synteny breaks in clade 2 occur near centromeres, consistent with the model that the clustering of centromeres favors recombination between pericentromeric sequences (1).

Another topic for future investigation is whether the type of DNA sequence underlying a centromere affects its function. Budding yeast display a variety of centromere types (10) (Fig. 1). Some clades have stable centromere organization and position, such as the *Saccharomycetaceae* family with point centromeres and the *Candida*/*Clavispora* clade with small regional centromeres. In other clades, centromeres evolve more rapidly. For example, the small regional centromeres in C. albicans are proposed to have arisen recently because they differ from the centromeres in related species (14). It will be interesting to learn whether some types of centromeres are associated with more aneuploidies and chromosomal rearrangements, which could facilitate adaptation to stress. If so, these types of centromeres might be favored in species that are primarily diploid and can better withstand chromosome loss.

In summary, by examining centromeres across the *Clavispora*/*Candida* clade, the Sanyal lab has uncovered their remarkable positional stability. They have also elucidated several centromere-adjacent rearrangements that could be associated with new traits, although these rearrangements do not account for the recent emergence of C. auris. Thus, this work sets the stage for studying the molecular mechanisms behind centromere stability and plasticity.
